# Evidence for glial reactivity using positron-emission tomography imaging of translocator Protein-18 kD [TSPO] in both sham and nerve-injured rats in a preclinical model of orofacial neuropathic pain

**DOI:** 10.1016/j.ynpai.2024.100175

**Published:** 2024-12-12

**Authors:** Gaelle M. Emvalomenos, James W.M. Kang, Sabrina Salberg, Crystal Li, Bianca Jupp, Matthew Long, Mohammad B. Haskali, Sunil Kellapatha, OIivia I. Davanzo, Hyunsol Lim, Richelle Mychasiuk, Kevin A. Keay, Luke A. Henderson

**Affiliations:** a School of Medical Sciences [Neuroscience], and the Brain & Mind Centre, The University of Sydney, NSW, 2006, Australia; b Department of Neuroscience, Central Clinical School, Monash University, Melbourne, Victoria, Australia; c The Radiopharmaceutical Research Laboratory, The Peter MacCallum Cancer Centre, Melbourne, Victoria, 3000, Australia

**Keywords:** Microglia, Trigeminal ganglion, Spinal trigeminal nucleus, Positron emission tomography, Chronic constriction injury, Macrophages

## Abstract

Chronic neuropathic pain is a debilitating condition that results from damage to the nervous system. Current treatments are largely ineffective, with limited understanding of the underlying mechanisms hindering development of effective treatments. Preclinical models of neuropathic pain have revealed that non-neural changes are important for the development of neuropathic pain, although these data are derived almost exclusively from post-mortem histological analyses. Although these static snapshots have provided valuable data, they cannot provide insights into non-neural cell changes that could be also assessed in human patients with chronic pain. In this study we used translocator protein 18 kDa (TSPO) PET imaging with [^18^F]PBR06 to visualise in-vivo, the activity of macrophages and microglia in a rodent preclinical model of trigeminal neuropathic pain. Using chronic constriction injury of the infraorbital nerve (ION-CCI) we compared temporal changes in TSPO binding in male rats, prior to, and up to 28 days after ION-CCI compared with both sham-injured and naïve counterparts. Unexpectedly, we found significant increases in TSPO signal in both ION-CCI and sham-injured rats within the trigeminal ganglion, spinal trigeminal nucleus and paratrigeminal nucleus during the initial phase following surgery and/or nerve injury. This increased TSPO binding returned to control levels by day 28. Qualitative histological appraisal of macrophage accumulation and glial reactivity in both ION-CCI and sham-injured rats indicated macrophage accumulation in the trigeminal ganglion and microglial reactivity in the brainstem trigeminal complex. These findings show, glial changes in the peripheral nerve and brain in both nerve-injured and sham-injured rats in a preclinical model of neuropathic pain which provides a platform for translation into human patients.

## Highlights

• Neuropathic pain treatments ineffective due to lack of mechanistic understanding.
• Cross-sectional preclinical histology reveals importance of non-neural cells.
• PET imaging reveals transient macrophage accumulation in the trigeminal ganglion.
• PET imaging reveals transient microglial reactivity in the brainstem.
• These PET imaging findings can be translated into humans with neuropathic pain.

## Introduction

Chronic orofacial neuropathic pain can arise from damage to peripheral branches of the trigeminal nerve. Such injuries often result in striking changes in sensitivity to both tactile and noxious stimuli applied to the head and orofacial regions, as well as intense episodic or persistent pain (International Classification of Orofacial Pain, 2020, Sessle, 2021). Trigeminal neuropathic pain is described as particularly distressing and intensely disabling, and most current treatments are largely ineffective over the long term (Haviv et al., 2017, Shueb et al., 2015, Smith et al., 2013). The complexity of neural changes triggered by damage to peripheral nerves underlies the significant failures in the long-term management of most neuropathic pain conditions. Preclinical models of neuropathic pain play an important role in simplifying this complexity and are contributing significantly to defining the neural and non-neural changes triggered by nerve damage.

Transection, compression, or constriction of either maxillary (*infraorbital n.*) or mandibular (*inferior alveolar n.* or *mental n.*) branches of the trigeminal nerve or damage to the trigeminal ganglion in the rat have each provided clinically relevant models of trigeminal neuropathy (Iwata et al., 2011, Sadighparvar et al., 2023, Krzyzanowska and Avendaño, 2012). Injury of a peripheral nerve triggers immediate changes at the site of damage, an immediate inflammatory response is mounted. The damaged axons and their associated Schwann cells release inflammatory mediators such as NGF and TNF-⍺, and immune cells, usually circulating macrophages migrate to the site, attracted by monocyte chemoattractant protein-1 (MCP-1), followed by invasion of the disrupted structures by these cells. These macrophages are usually a mixture of both pro- and anti-inflammatory phenotypes (Komori et al., 2011, Donegan et al., 2013, Shinoda et al., 2022). This process often results in an increased firing of the nerve both spontaneously and in response to activation of its sensory terminals (Gudes et al., 2015, Kanamori et al., 2007, Leo et al., 2015, Peng et al., 2016). Once established, these processes trigger changes along both damaged and undamaged nerve fibres as they project towards their central nervous system targets, i.e., the chief sensory nucleus of the trigeminal, as well as along the trigeminal tract to the spinal nucleus (SpV), and the dorsal horn of the upper cervical spinal cord (UCC).

In response to injury to a branch of the trigeminal nerve, the ganglion also shows injury related changes, both resident and infiltrating macrophages are reported to accumulate within the division of the ganglion through which the damaged nerves pass, and where their damaged cell bodies reside (Komori et al., 2011, Donegan et al., 2013, Iwai et al., 2021, Guimarães et al., 2023). This macrophage invasion and proliferation is driven both by increased cytokines, for example chemokine C-C motif ligand 2 (CCL2) released from damaged ganglion cells, as well as by the barrages of spontaneous activity triggered by the damaged fibres (Zhu et al., 2014, Liu et al., 2016, Kim et al., 2011). Satellite glial cells are reported to show activated states in some reports and begin to express gap junctions, specifically connexin-43, which facilitates glial-glial communication, the result of which drives increased activity in ganglionic cells (Kaji et al., 2016). This increase in neural activity is also enhanced by the release of TNF-⍺ from the infiltrating macrophages (Batbold et al., 2017).

The ganglionic changes then drive further change in brainstem recipient regions specifically in the SpV and the UCC, a large portion of the SpV shares a similar laminar architecture to the UCC and is the region that receives most of the trigeminal nociceptive input. This region sends projections to higher brainstem and thalamic pain processing regions, including the parabrachial region, the midbrain periaqueductal gray matter (PAG), hypothalamus, amygdala, medial thalamus as well as the medial part of the ventroposterior thalamus (Saito et al., 2017, Okada et al., 2019). Neuronal activity in these regions has been shown to change in preclinical models of trigeminal neuropathic pain (Okada et al., 2019, Dubner and Ren, 2004, Vos and Strassman, 1995). Microglia are the “resident macrophages” of the central nervous system, although occasionally, circulating macrophages can enter the parenchyma, including the brainstem. Trigeminal nerve injuries are reported to trigger the activation of microglia within the SpV, and they may also contribute significantly to the pain sensations in the immediate injury phase (Okada-Ogawa et al., 2009, Piao et al., 2006, Asano et al., 2020, Hayashi et al., 2023). Microglia share similar roles to macrophages, one of which is the production of cytokines that have pro-inflammatory effects (for example TNF-⍺, BDNF, IL-1b, IL-6). These cytokines act via several different mechanisms to increase neuronal excitation (Coull et al., 2005, Guo et al., 2007, Kawasaki et al., 2008, Hildebrand et al., 2016, Zhou et al., 2019). This sustained neural activity is believed to contribute significantly to the pain of trigeminal nerve injury. Microglial activity can be sustained over time (Clark et al., 2007) and in principle could contribute to the chronicity of pain, however inhibition of microglial activity has not shown consistent or substantial successes in attenuating ongoing pain in preclinical models (Piao et al., 2006, Zama et al., 2019, Baggio et al., 2023).

Microglial activation drives activity in co-located astrocytes, thus trigeminal nerve injury is also associated with significant changes in astrocyte activity in the same anatomical regions; importantly modulation of astrocyte activity appears to have a stronger attenuation on injury evoked pain (Okada-Ogawa et al., 2009, Tsuboi et al., 2011, Dieb and Hafidi, 2013). It is clear that recruitment and activation of macrophages, microglia, and astrocytes, at the site of injury, in the sensory ganglion and the brainstem nuclei, plays a critical role in the establishment of neuropathic pain by increasing neural excitability (Iwata and Sessle, 2019). These findings of non-neuronal contributions to the development of neuropathic pain are derived almost exclusively from post-mortem histological/immunohistochemical analyses. In this study we used translocator protein 18 kDa (TSPO) PET imaging with [^18^F]PBR06 to visualise *in-vivo*, the activity of non-neuronal cells (macrophages and glia) following trigeminal nerve injury in the rat. TSPO ligands have been used successfully for brain imaging in several human pain conditions, despite suggestions of low specificity (Alshelh et al., 2022, Loggia et al., 2015, Emvalomenos et al., 2024). This success highlights the great potential for use in preclinical models, thus enhancing translational opportunities, which includes identifying and understanding specific cellular contributions to the PET signal (Emvalomenos et al., 2024). Using chronic constriction injury of the infraorbital nerve (ION-CCI) (Vos et al., 1994) our aim was to describe temporal changes in TSPO binding in male rats, prior to, and up to 28 days after ION-CCI compared to sham-injured and naïve counterparts. Sites of significant TSPO signal change were evaluated qualitatively for evidence of macrophage accumulation and glial reactivity in the infraorbital nerve, trigeminal ganglion, and the brainstem trigeminal complex.

## Materials and Methods

All experimental procedures were carried out with the approval of Alfred Medical Research and Education Precinct Animal Ethics Committee at the Precinct Animal Centre (PAC; E/8283/2022/M) and in accordance with the guidelines of the Code for the Care and Use of Animals in Research Australia, and Ethical Guidelines for Investigation Association for the Study of Pain (Zimmermann, 1983).

### Experimental Design

Male Sprague-Dawley rats (n = 79) were sourced from the Monash Animal Research Platform and kept for the duration of the experiment on a 12:12hr light:dark cycle in a temperature controlled environment. Rats at the transition from adolescence (6 weeks) to adulthood were used in these studies to ensure the optimal range of head size for the scanners over the duration of the study. Rats arrive at the facility at 6 weeks of age, and habituate for at least 1 week. Animals were housed 2–3 per cage with *ad libitum* access to standard laboratory chow and water.

At seven weeks of age, rats were arbitrarily allocated to one of three groups: (i) infraorbital nerve chronic constriction injury (ION-CCI) (n = 35), (ii) sham surgical procedures (sham) (n = 23), or (iii) uninjured controls (naïve) (n = 21). The ION-CCI and sham surgical rats were then assigned to one of four imaging time points post ION-CCI or sham surgery: 2 days (**D2**) (sham n = 6; ION-CCI n = 8), 7 days (**D7**) (sham n = 5; ION-CCI n = 9), 14 days (**D14**) (sham n = 7, ION-CCI n = 10), or 28 days (**D28**) (sham, n = 5; ION-CCI n = 8). The naïve rats (n = 21) were placed into a single group for comparison with the ION-CCI and sham groups at each timepoint. On their allocated imaging day, each rat was scanned once for approximately 60 min in a 9.4 Tesla Bruker MRI scanner and then transferred to a PET/CT scanner for further imaging. On completion of the PET/CT scan, rats were permitted to recover from anaesthesia. Two days later, the naïve rats and the ION-CCI and sham rats at each time point (**D2-D28**) were culled and their tissues were collected.

### Surgical Procedures: Infraorbital nerve constriction injury (ION-CCI)

Rats were anaesthetised by induction with isoflurane (4 % in 100 % oxygen), delivered via an airtight induction chamber. Surgical level anaesthesia was maintained with 1.5–2 % isoflurane in 100 % oxygen administered via a custom-made facemask. Body temperature was maintained by a thermal blanket. ION-CCI was performed as described by Vos and colleagues (Vos et al., 1994). The following procedures were performed under x3.5 magnification using Galilean Loupes (28050–35 Fine Scientific Tools, Vancouver Canada). The midline above the nasal bones and between the eyes was shaved and sterilised with povidone-iodine. Once the blink reflex and pedal reflex were abolished, an incision was made approximately 12 mm long, and 2 mm medial to the supraorbital ridge of the right orbit, following the curvature of the frontal bone. The fascia and muscle were gently teased from the bone using blunt dissection until the contents of the orbit could be gently retracted laterally. Following retraction of the orbital contents, the ION was visualised approximately 8 mm deep within the orbit, lying within the infraorbital canal of the maxillary bone. Once visualised, 5 mm of the ION was gently freed from the surrounding connective tissue via blunt dissection. Two chromic gut ligatures (Chromic Gut 5–0 sutures, #687G, Ethicon, Inc.) were loosely tied around the ION using a square knot, tight enough to slightly depress the nerve, but not enough to occlude epineural circulation. The ligatures were spaced approximately 2 mm apart, and the ION was repositioned in the infraorbital canal of the maxillary bone. The incision was sutured closed with nylon sutures (Nylon Sutures 7–0, #1696G, Ethicon Inc.). The incision site was cleaned, and antibiotic powder was dusted over the suture site. The rat was moved to a heated recovery cage and monitored closely until ambulatory and eating and drinking. Sham surgical procedures were conducted identically, the nerve was isolated and exposed for the same duration as the application of the ligatures in the nerve-injured group, but no ligatures were applied to the ION.

### Imaging procedures

All procedures were performed at the Alfred Research Alliance-Monash Biomedical Imaging (ARA-MBI) facility (Alfred Centre, Melbourne, Australia). PET/CT imaging was performed using a Mediso NanoScan PET/CT (Mediso Ltd., Hungary). The [^18^F]PBR06 was synthesized at the Peter MacCallum Cancer Centre with the radiochemical purity of [^18^F]PBR06 always exceeding 95 %, and the molar activity ∼ 18,500 MBq/µmol. A maximum of six animals were imaged per imaging session at the different endpoints, **D2** to **D28** as described above. Rats were anaesthetised with isoflurane for the duration of all scanning, they were induced in an air-tight box (3–4 %), then transferred to a custom-built facemask, where isoflurane level was maintained at 1–3 %. Each rat then underwent a series of MRI scanning sequences for 1 h and 15 min prior to be transferred to the PET/CT (animal kept under anaesthesia for the whole process). Animals were placed into the PET/CT in a supine position, headfirst, with the brain and heart in the field of view (FoV). At the start of the dynamic PET acquisition, animals were injected via the dorsal penile vein with a bolus of 0.1987 mCi to 0.6343 mCi of [^18^F]PBR06 in volumes ranging from 0.04 to 0.45mls. PET scans were acquired continuously for a total of 60 min with 1–3 coincidence mode, a coincidence time-window of 5 ns and an energy window of 400–600 keV. Following the PET scan, two CT scans were acquired over a period of 10 min. The first CT image was acquired using the same field of view as the PET and was used for attenuation correction (70 kVp at 700 µA, 480 projections per bed position, helical mode, scan length of 98.2 mm, 300 ms exposure time and 1:4 binning). The second CT image covered the head only and was used for co-registration (70 kVp at 700 µA, 720 projections per bed position, semi-circular single FoV mode, with medium zoom, scan length of 37.1 mm, 300 ms exposure time and 1:1 binning).

### PET image processing and analysis

The raw PET list-mode data was reconstructed using the Mediso software, Tera-Tomo 3D reconstruction method, 2 iterations of a 3-dimensional ordered subsets expectation maximization (3D OSEM) algorithm (6 subsets) and with a matrix size of 142x142x159, an isotropic voxel size of 0.6 mm and with the following corrections, attenuation, scatter, random and ^18^F decay. The length of the dynamic scan frames were 5x1min, 5x2min, 3x5min, 3x10min. Time-activity-curves were obtained to assess the kinetics of the [^18^F]PBR06 radiotracer in the brain. The last 30 min, corresponding to the relatively stable transient equilibrium, were used to calculate the Standard Uptake Values (SUVs_30-60min_), which is SUV_30-60min_ body weight [g/ml] = (Tissue activity/Decay corrected injected dose)*Weight*1000. To account for the amount of radiotracer that crossed the blood–brain barrier, these SUV values were divided by the whole brain SUV for each rat to obtain the SUV ratio (SUVr_30-60min_) (Alshelh et al., 2022, Wimberley et al., 2021). PET images were then pre-processed using Amide’s A Medical Image Data Examiner (Loening and Gambhir, 2003), Statistical Parametric Mapping 12 (SPM12) and in-house Matlab scripts (Matlab, Mathworks, R2023a). CT and SUV and SUVr images were co-registered to the Waxholm Space atlas of the Sprague Dawley rat brain (Papp et al., 2014). These co-registered brain maps were then resliced at 0.3x0.3x0.3 mm voxel size and smoothed using a 0.9 mm full-width-at-half-maximum (FWHM) Gaussian kernel.

To determine significant differences between groups, using SPM12, voxel-by-voxel comparisons were made using the co-registered, resliced, and smoothed SUV and SUVr brain images. Our primary goal was to determine the effects of ION-CCI over time on TSPO binding, we compared ION-CCI groups at each timepoint (D2, D7, D14, D28) with naïve and sham-injured rats. There were no significant differences between ION-CCI rats and sham-injured rats, therefore we analysed each group compared to naïves. Significant differences in TSPO binding (SUVr brain maps) between groups were determined using second level, two sample, random effects analyses (p < 0.05, family wise error [FWE] corrected for multiple comparisons). Because there were no differences between ION-CCI and sham groups, for each significant cluster derived from the ION-CCI versus naïve analysis at a particular time point, we extracted the SUVr values from the naïve group and the ION-CCI and sham groups at each timepoint and the mean (±SEM) was calculated and plotted. Significant differences between groups then were determined between groups using two-sample t-tests (p < 0.05, Bonferroni corrected for multiple comparisons). Comparisons between ION-CCI and sham groups did not reveal significant differences between nerve-injured and sham-injured groups.

Since we hypothesised that changes in glial reactivity would occur along the ascending trigeminal pain pathway, we restricted this initial analysis to the right (ipsilateral to injury) spinal trigeminal nucleus (SpV), left ventroposterior medial thalamus (VPM) and left primary somatosensory cortex (S1). These masks were created using the volume-of-interest (VOIs) masks within the Waxholm Space atlas of the Sprague Dawley rat brain. To further explore changes in SpV, we also created effect size maps for each animal and then created a mean effect size map for each ION-CCI group and for the naïve group. We created effect size difference maps by subtracting each ION-CCI group from the naïve group, masked the right (ipsilateral to injury) SpV, and overlaid the resultant SpV maps onto a rat brain template for each of the four timepoints. In addition, we conducted a non-masked voxel-by-voxel analysis of the brain to determine significant increases or decreases in TSPO binding between ION-CCI groups at each time point and the naïve group. Since we found no significant differences at FEW corrected levels, we used a more liberal threshold of p < 0.001, uncorrected to assess more subtle group differences. To limit the potential for Type 1 errors we used a minimum cluster size of 20 contiguous voxels.

In addition to exploring TSPO binding changes in the brain, we hypothesized that in the ION-CCI groups, the injured trigeminal nerve/ganglion would also display significantly increased TSPO binding. For each timepoint, significant differences in TSPO binding (SUV maps) between the ION-CCI groups and naïve group were determined using second level, two sample, random effects analyses. We restricted this initial analysis to the right trigeminal nerve/ganglion using a custom mask. Since we found no significant differences at FWE corrected levels, we used a liberal threshold of p < 0.05, uncorrected to assess more subtle group differences. To limit the potential for Type 1 errors we used a minimum cluster size of 20 contiguous voxels. For each significant cluster at a particular time point, SUV values were extracted for all ION-CCI, sham and naïve groups and the mean (±SEM) calculated and plotted. SUV was used for analysis of peripheral structures because of the relatively small volumes of the structures analysed, whereas for the much larger brain structures SUVr was used for analyses. Significant differences were determined between groups using two-sample *t*-test (p < 0.05, Bonferroni corrected for multiple comparisons). Comparison between ION-CCI and sham groups did not reveal significant differences between nerve-injured and sham-injured groups.

### Euthanasia and Perfusion

Forty-eight hours following the imaging session, animals were briefly anaesthetised with 5 % isoflurane mixed in oxygen 1.5 L/minute and a lethal dose of pentobarbitone administered (130 mg/kg, intraperitoneally). Each rat was then perfused *trans*-cardially with 400 ml of ice cold heparinised 0.9 % (w/v) saline followed by 400 ml of 4 % paraformaldehyde in sodium acetate-borate buffer (pH 9.6, 4 °C) (PFA). The left and right infraorbital nerves (ION), their corresponding trigeminal ganglion (TG) and the brain and cervical spinal cord were dissected free and post-fixed in PFA for two hours at room temperature, then cryoprotected in 10 % (w/v) sucrose in 0.1 M phosphate buffered saline, pH 7.4 (PBS) and stored at 4 °C until processing.

### Qualitative Histological Verification

All tissue was sectioned using a cryostat (LEICA CM1950, Germany), a 1 in 6 series of transverse sections of the infraorbital nerve (14 µm); and the trigeminal ganglia (14 µm) were cut onto 2 % (w/v) gelatinised slides and stored at -20 °C until processing. The medulla was blocked caudally with a coronal cut at approximately the C3 level of the cervical spinal cord, and rostrally at the level of the subnucleus interpolaris of the SpV (−12.00 mm bregma (Paxinos and Watson, 2014). Coronal sections of the medulla were cut at 40 µm, as a 1 in 10 series and stored in antifreeze at −20 °C until processed.

ION, TGs, and sections of medulla were immunoreacted to determine the co-localisation of CD68-immunoreactive (CD68-IR) macrophages, Peripheral-Type Benzodiazepine Receptor (PBR) immunoreactivity (PBR-IR), blood vessels (tomato lectin immunoreactivity [TL + ]), ionized calcium binding adaptor molecular 1 (IBA1) immunoreactive microglia, or Glial Fibrillary Acidic Protein (GFAP) immunoreactive (GFAP-IR) astrocytes.

### Infraorbital nerve and trigeminal ganglion

ION and TG mounted slides were washed in 0.1 M phosphate buffered saline, pH7.4 (PBS) and blocked in PBS containing 5 % (v/v) normal horse serum (NHS) and 0.05 % (v/v) Tween-20 (Sigma-Aldrich, Australia). Sections were incubated for 16-hours at room temperature with mouse anti-CD68 [ED-1] (1:250, Abcam, Melbourne, Vic, Australia, RRID: AB_1141557) and rabbit anti-PBR [EPR5384] (1:100, Abcam, Melbourne, Vic, Australia, RRID: AB_10862345), in PBS containing 5 % (v/v) NHS and 0.05 % (v/v) Tween-20. Sections were then washed in PBS and incubated for 2-hours in Alexa-488 conjugated donkey anti-mouse (1:500, RRID: AB_2340846, Jackson, West Grove, PA, USA) and Cy3 conjugated Donkey anti-rabbit (1:500, RRID: AB_2307443, Jackson, West Grove, PA, USA). Sections were washed in PBS and then incubated for 3-hours with DyLight™ 594 conjugated Tomato [Lycopersicon Esculentum] Lectin (TL) in PBS (1:1000, RRID: AB_2336416, Vector Laboratories, Inc, Newark, CA, USA). Slides were washed in PBS and then incubated for 20-mins with DAPI (1:20, ThermoFisher Scientific, RRID: AB_2307445) in PBS. Slides were washed in PBS before being cover slipped with Prolong Gold Antifade (P36931, Invitrogen) mounting medium and stored at 4 °C until microscopy.

### Medulla

Medulla sections were washed in PBS and then blocked in 5 % (v/v) NHS in PBS for 30-minutes at room temperature. *PBR/GFAP/TL immunofluorescence:* Sections were incubated for 16-hours at 4 °C with rabbit anti-PBR [EPR5384] (1:100, RRID: AB_10862345, Abcam, Melbourne, Vic, Australia,) and mouse anti-GFAP (1:5000, RRID: AB_477010, Merck Pty Ltd., Sydney, NSW, Australia). Sections were then washed in PBS and incubated for 2-hours in Alexa-488 conjugated donkey anti-mouse (1:500, RRID:AB_2340846, Jackson, West Grove, PA, USA) and Cy3 conjugated donkey anti-rabbit (1:500, RRID:AB_2307443, Jackson, West Grove, PA, USA). *PBR/IBA1/TL immunofluorescence:* Sections were incubated for 16-hours at 4 °C with rabbit anti-PBR [EPR5384] (1:100, Abcam, Melbourne, Vic, Australia, RRID: AB_10862345), sections were then washed in PBS and incubated for 2-hours in Cy3 conjugated donkey anti-rabbit (1:500, RRID:AB_2307443, Jackson, West Grove, PA, USA). Sections were washed and then incubated for 16-hours at 4 °C with rabbit anti-IBA1 (1:1000, RRID: AB_2636859, Abcam, Melbourne, Vic, Australia), washed in PBS and incubated for 2-hours in Alexa-488 conjugated donkey anti-rabbit (1:500, RRID: AB_2313584, Jackson, West Grove, PA, USA). Following antibody incubations all sections were then washed in PBS and incubated for 3-hours with DyLight™ 594 conjugated Tomato [Lycopersicon Esculentum] Lectin (TL) in PBS (1:1000, RRID: AB_2336416, Vector Laboratories, Inc, Newark, CA, USA). Slides were washed in PBS and then incubated for 20-mins with DAPI (1:20, ThermoFisher Scientific, RRID: AB_2307445) in PBS. Slides were washed in PBS before being mounted and cover slipped with Prolong Gold Antifade (P36931, Invitrogen) mounting medium and stored at 4 °C until analysed under the microscope.

### Microscopy

Slides were imaged using a Nikon C2 confocal microscope. The large image function was used to ‘stitch’ multiple images (100x magnification) to allow the visualisation of the entire infraorbital nerve including ligated areas, trigeminal ganglia, and coronal medulla sections. Representative z-stack images were captured at 600x magnification to visualise co-localisation of CD68-IR macrophages, PBR-IR, TL + blood vessels, IBA1-IR microglia, or GFAP-IR astrocytes.

## Results

### Trigeminal Pain Pathway: Trigeminal Ganglion

**Fig. 1:**
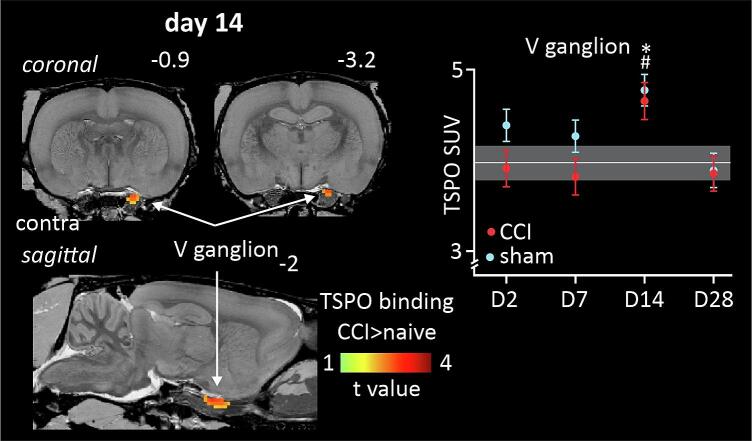
TSPO binding assessed in infra-orbital nerve/ganglion in chronic constriction injury (ION-CCI), sham-injured and naïve groups. Significant binding increases (hot colour scale) at day 14 (D14) in the ION-CCI compared with naive groups occurred in the region encompassing the right (ipsilateral to nerve injury) trigeminal (V) ganglion. Clusters are overlaid onto a T2-weighted rat brain template anatomical image set. To the top right of each image is the approximate location relative to bregma derived from Paxinos and Watson (2018). To the right are plots of mean ± SEM standard uptake value (SUV) for all three groups at day 2 (**D2**), day 7 (**D7**), day 14 (**D14**) and day 28 (**D28**). The horizontal grey line and grey shading indicates the mean and SEM of the naïve group. *p < 0.05 derived from voxel-by-voxel analysis, # p < 0.05 post hoc two-sample t-tests sham versus naïve (values extracted from significant clusters derived from ION-CCI vs naïve voxel-by-voxel analysis).

**Fig. 2:**
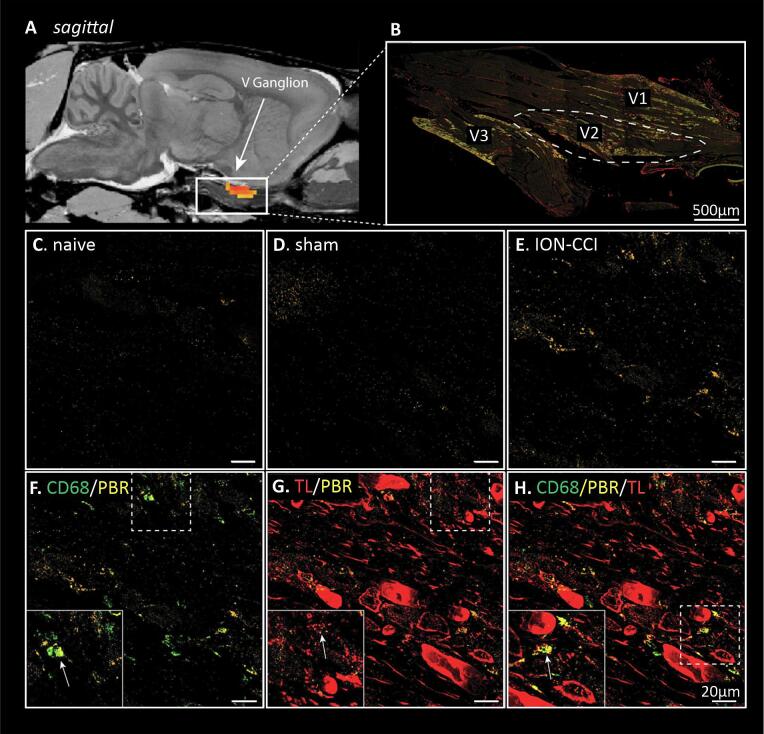
Representative photomicrographs of PBR-IR co-localization. **A:** trigeminal (V) ganglion TSPO binding cluster overlaid on a T2-weighted rat brain template anatomical image. **B:** stitched 40X magnification photomicrograph of the trigeminal ganglion and corresponding ophthalmic (V1), maxillary (V2) and mandibular (V3) divisions with PBR-IR (orange), CD68-IR (green) and TL (red) co-localization with immunofluorescence. The PBR-IR in the V2 of **(C)** naïve, **(D)** sham-injured and **(E)** ION-CCI rats are shown at 600X magnification. Example of co-localization of **(F)** CD68-IR and PBR-IR, **(G)** TL + and PBR-IR and **(H)** CD68-IR, PBR-IR and TL + . White arrows show co-localized immunofluorescent signals. Scale bars represent 20 µm or 500 µm in panel B. (For interpretation of the references to colour in this figure legend, the reader is referred to the web version of this article.)

**Supplementary Fig. 1:**
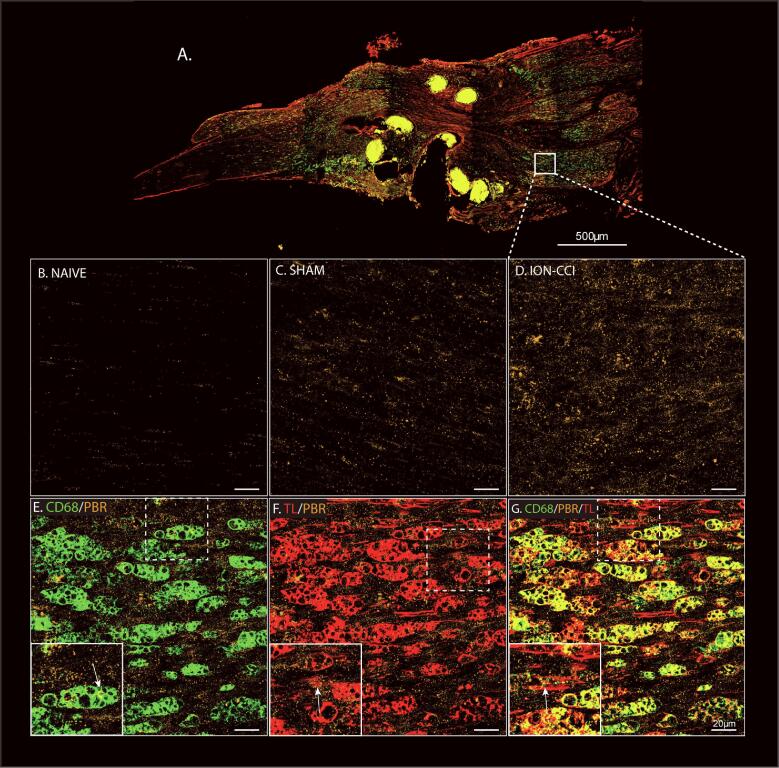


Analysis of the trigeminal ganglion and nerve revealed that relative to the naïve group, there was a significant increase in binding in the trigeminal ganglion ipsilateral to the nerve constriction in the ION-CCI rats at day 14, and no differences at days 2, 7 or 28 (Fig. 1, Table 1). A similar increase was observed also in the sham-injured rats at this time point (Fig. 1), although the TSPO binding was somewhat higher in sham-injured rats, at days 2 and 7 post-ION-CCI, this was not significantly different. Qualitative histological observations of the ganglion of ION-CCI rats revealed PBR-IR in the maxillary division of the ganglion co-localized with CD68-IR macrophages, and TL + endothelial cells of the ganglionic vasculature (Fig. 2E-H). In ganglia from sham-injured rats, PBR-IR was also observed (see Fig. 2D). Naïve rats showed little PBR-IR. At the site of injury, PBR-IR was frequently colocalized with CD68-IR macrophages and TL + endoneurial vasculature (Supp Fig. 1).

**Table 1:** Significant cluster properties including SUVr values in the ascending trigeminal pathway and SUV values for the trigeminal ganglion. * p < 0.05 voxel-by-voxel analysis. # p < 0.05, two sample *t*-test. Day 2: day 2; D7: day 7; D14: day 14; D28: day 28; ION-CCI: infra-orbital nerve chronic constriction injury; SEM: standard error mean; SpV: spinal trigeminal nucleus; SUV: standard uptake values; SUVr: standard uptake values ratio.

Ascending trigeminal pathway: ION-CCI > naive	cluster size	t- value	Day		SUVr mean ± SEM	
				naive	sham-injured	ION-CCI
D2: SpV cluster	8	3.27	D2		1.77 ± 0.03^#^	1.80 ± 0.08*
			D7	1.59 ± 0.03	1.65 ± 0.07	1.80 ± 0.07^#^
			D14		1.54 ± 0.09	1.70 ± 0.05^#^
			D28		1.47 ± 0.08	1.53 ± 0.03
D7: SpV cluster	53	3.91	D2		1.79 ± 0.03^#^	1.79 ± 0.07^#^
			D7	1.61 ± 0.03	1.69 ± 0.06	1.84 ± 0.06*
			D14		1.57 ± 0.09	1.72 ± 0.05^#^
			D28		1.48 ± 0.07	1.52 ± 0.04
Trigeminal ganglion/nerve: ION-CCI > naive					SUV mean ± SEM	
D14: trigeminal ganglion cluster	116	2.31	D2		4.38 ± 0.37	3.90 ± 0.30
			D7	3.96 ± 0.19	4.25 ± 0.35	3.81 ± 0.19
			D14		4.75 ± 0.17^#^	4.64 ± 0.23*
			D28		3.90 ± 0.34	3.84 ± 0.27

### Trigeminal Pain Pathway: Brainstem Trigeminal Complex:

**Fig. 3:**
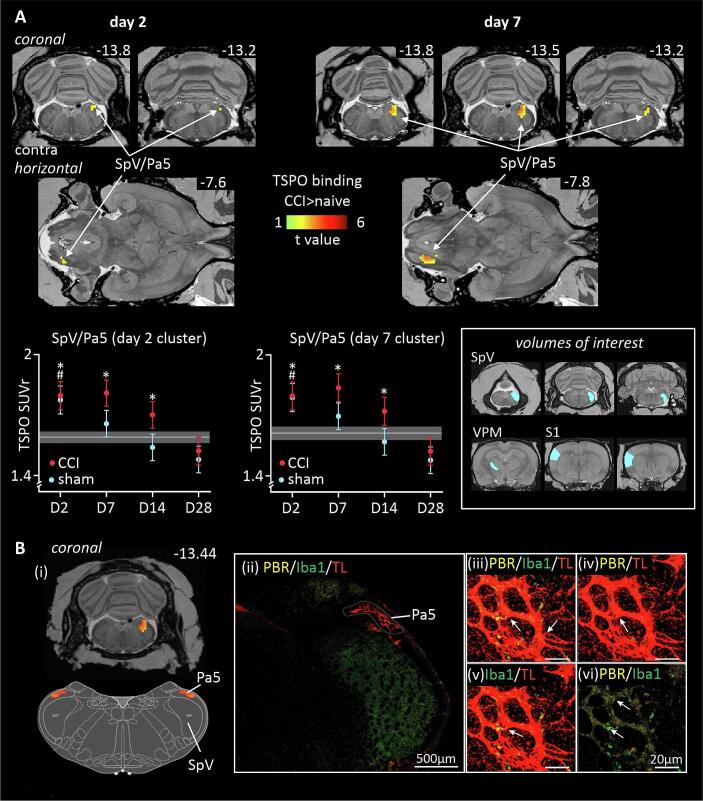
TSPO binding assessed in the ascending trigeminal pathway in infra-orbital nerve chronic constriction injury (ION-CCI), sham-injured and naïve groups. **A**: Significant binding increases (hot colour scale) at day 2 (**D2**) and day 7 (**D7**) in the ION-CCI compared with naive groups. Binding increases occurred in the right (ipsilateral to nerve injury) brainstem trigeminal complex. Clusters are overlaid onto a T2-weighted rat brain template anatomical image set. To the top right of each image is the approximate location relative to bregma derived from a rat atlas Paxinos and Watson (2018). Below the overlays are plots of mean ± SEM standard uptake value ratios (SUVr) for all three groups. The horizontal grey line and grey shading indicates the mean and SEM of the naïve group. *p < 0.05 derived from voxel-by-voxel analysis, # p < 0.05 post hoc two-sample t-tests sham versus naïve (values extracted from significant clusters derived from ION-CCI vs naïve voxel-by-voxel analysis). Inset to the right shows the volumes of interest (light-blue) used for analysis. **B:** Representative photomicrographs of PBR-IR co-localization within the Brainstem Trigeminal Complex. (i) TSPO binding cluster overlaid on a T2-weighted rat brain template anatomical image of the brainstem trigeminal complex at approximately −13.44 mm bregma (ii) stitched 40x magnification photomicrograph of the brainstem trigeminal complex, with the paratrigeminal nucleus (Pa5) outlined in white and PBR-IR (orange), Iba1-IR (green) and TL (red) co-localization with immunofluorescence. Example of co-localization of (iii) PBR-IR, Iba1-IR and TL+; (iv) PBR-IR and TL+; (v) Iba1-IR and TL+; and (vi) PBR-IR and Iba1-IR. White arrows depict co-localized immunofluorescent signals. Scalebars represent 20 µm or 500 µm in panels ii-vi. (For interpretation of the references to colour in this figure legend, the reader is referred to the web version of this article.)

Analysis of TSPO binding in the brainstem trigeminal complex, revealed significant increases in the SpV/paratrigeminal region (Pa5) of the ION-CCI group compared to the naïve group at day 2 and day 7 (Fig. 3A). Increased binding was restricted to the right Pa5. Extraction of SUVr values from the significant Pa5 cluster at day 2, revealed that compared with the naïve group, ION-CCI groups displayed greater TSPO binding at days 2, 7 and 14, but not at day 28 (Table 1). Sham-injured rats showed a significant increase in TSPO binding compared to the naïve group at day 2, but not at days 7, 14 and 28. Once again, extraction of SUVr values from the significant Pa5 cluster at day 7, revealed that compared with the naïve group, ION-CCI rats had greater TSPO binding at days 2, 7 and 14, but not day 28. Sham-injured rats displayed a significant increase in TSPO binding compared with naïve group at day 2, but not at days 7, 14 and 28. Qualitative observations of the Pa5 region confirmed PBR-IR localised to this region which was usually co-localized with IBA1-IR microglia, and TL + endothelial cells in the vasculature, (Fig. 3B) but never in GFAP-IR astrocytes in the sections observed (not illustrated).

**Fig. 4:**
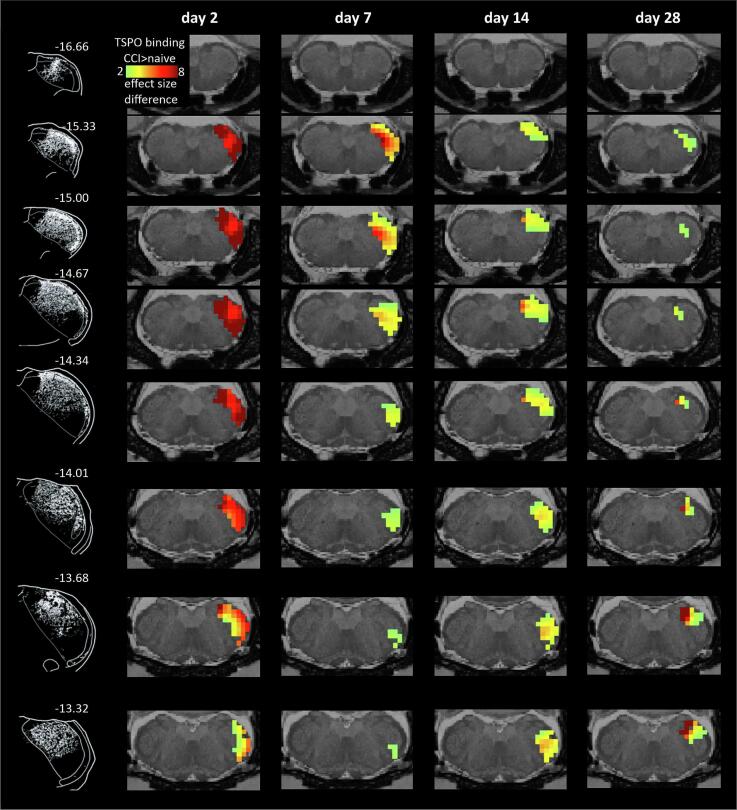
Effect size increases (hot colour scale) of TSPO binding in infra-orbital nerve chronic constriction injury (ION-CCI) compared with naïve groups within the right brainstem trigeminal complex volumes of interest at day 2, day 7, day 14 and day 28 overlaid onto coronal slices of a T2-weighted rat brain anatomical image set. On the left are shown the terminal projections of the infraorbital nerve traced using WGA- conjugated horse-radish peroxidase, the data are adapted from Fig. 4 of (Panneton et al., 2010). To the top right of each image is the approximate location relative to bregma derived from a rat atlas.

**Fig. 5:**
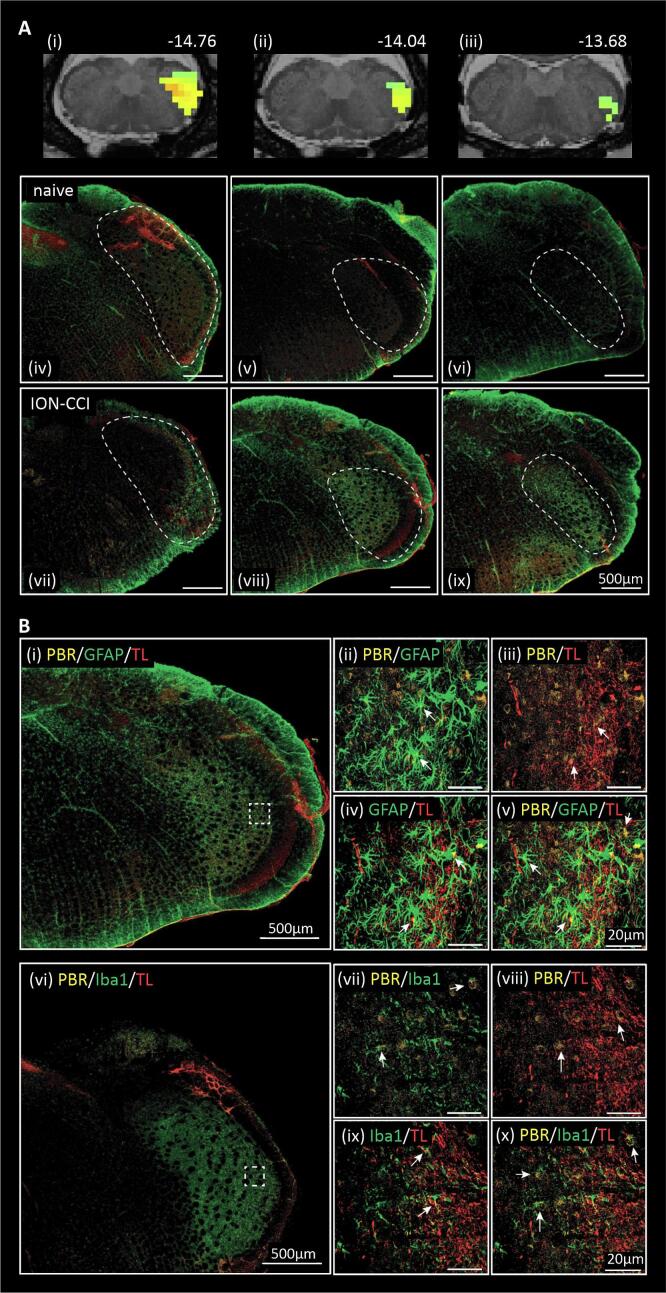
**A:** Top row shows significantly greater TSPO binding in the infra-orbital nerve chronic constriction injury (ION-CCI) compared with naïve groups at day 7 overlaid onto a series of coronal T2-weighted anatomical images between −13.68 mm and −14.76 mm relative to bregma. Below are photomicrographs of coronal sections of the brainstem trigeminal complex at equivalent levels stained for PBR-IR (orange), GFAP-IR (green) and TL (red) co-localization with immunofluorescence. The upper row of photomicrographs shows sections from naïve control rats and the lower row of rats following ION-CCI. All data are from rats at day 7 post-injury. Scale bar 500 µm. **B:** Photomicrographs of coronal sections of the brainstem trigeminal complex at day 7 post-injury. Top (i) large photomicrograph, stitched 40x magnification of the brainstem trigeminal complex, with high magnification insets (to right) taken from the region of the SpV outlined in white. PBR-IR (orange), GFAP-IR (green) and TL+ (red) co-localization with immunofluorescence. To the right, insets show examples of co-localization of: (ii) PBR-IR, & GFAP; (iii) PBR-IR & TL+; (iv) GFAP-IR & TL+; and (v) PBR-IR, GFAP-IR & TL + . White arrows depict co-localized immunofluorescent signals. Scalebars represent 20 µm or 500 µm. Bottom Panel: Left: large photomicrograph, stitched 40x magnification of the brainstem trigeminal complex, with high magnification insets (to right) taken from the region of the SpV outlined in white. PBR-IR (orange), Iba1-IR (green) and TL+ (red) co-localization with immunofluorescence. To the right, insets show examples of co-localization of: (a) PBR-IR, & Iba1-IR; (b) PBR-IR & TL+; (c) Iba1-IR −IR & TL+; and (d) PBR-IR, Iba1-IR −IR & TL + . White arrows depict co-localized immunofluorescent signals. Scalebars represent 20 µm or 500 µm. (For interpretation of the references to colour in this figure legend, the reader is referred to the web version of this article.)

Fig. 4 shows overlays of the mean effect size differences within the right SpV and shows increased TSPO binding across the rostro-caudal extent of the SpV at days 2–14 and by day 28, these increases remain only in the Pa5 region, at approximately −12.8 mm to 13.5 mm from bregma. Histological analysis of the SpV region confirmed PBR-IR specific to this region and was co-localized with IBA1-IR microglia, and TL + endothelial cells in the vasculature, and GFAP-IR astrocytes (Fig. 5A and B).

### Trigeminal Pain Pathway: Forebrain Structures:

**Fig. 6:**
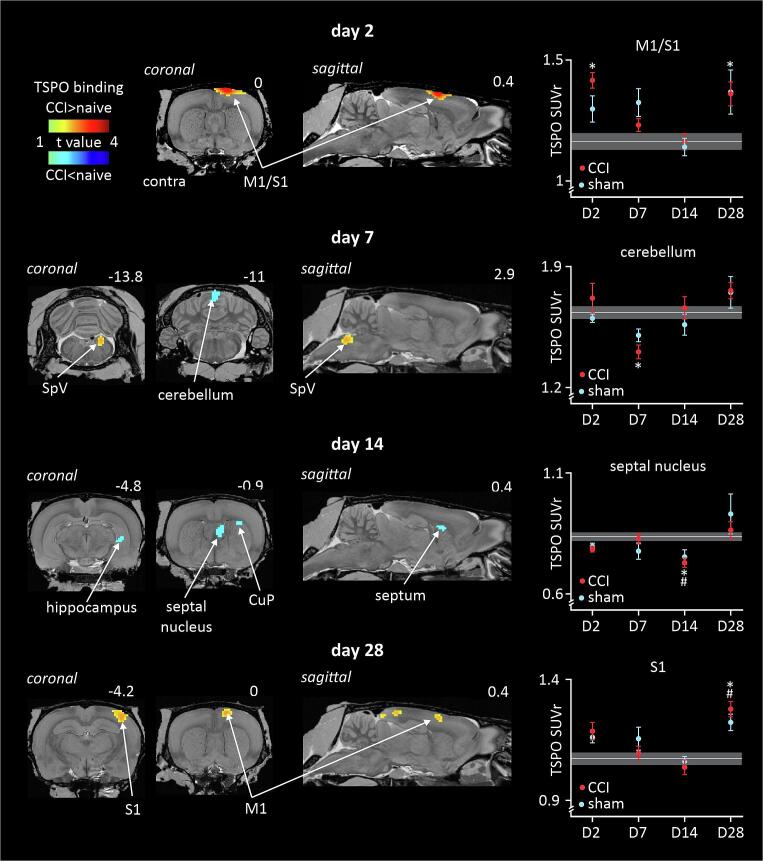
TSPO binding assessed over the entire brain in infra-orbital nerve chronic constriction injury (ION-CCI), sham-injury and naïve groups. Significant binding increases (hot colour scale) or decreases in ION-CCI compared with naïve groups at day 2 (**D2**), day 7 (**D7**), day 14 (**D14**) and day 28 (**D28**) are displayed. Clusters are overlaid onto a T2-weighted rat brain template anatomical image set. To the top right of each image is the approximate location relative to bregma derived from Paxinos and Watson (2018). To the right are plots of mean ± SEM standard uptake value ratios (SUVr) for all three groups. The horizontal grey line and grey shading indicates the mean and SEM of the naïve group. *p < 0.05 derived from voxel-by-voxel analysis, # p < 0.05 post hoc two-sample t-tests sham-injury versus naïve (values extracted from significant clusters derived from ION-CCI vs naïve voxel-by-voxel analysis). CuP: caudate/putamen; M1: primary motor cortex; S1: primary somatosensory cortex; SpV: spinal trigeminal nucleus; SUVr: standard uptake values ratio.

Analysis of supra-medullary brain regions revealed a number, of TSPO binding differences between ION-CCI and naïve rats within the 28-day period. On day 2, TSPO binding was significantly increased in the area encompassing both the ipsilateral primary motor cortex (M1) and primary somatosensory cortex (S1) (Fig. 6, Table 2). Extraction of SUVr values from the cluster located in this combined M1/S1 cortical region, revealed that compared with the naïve group, the ION-CCI rats also displayed greater TSPO binding at day 28 whereas the sham-injury group did not display a significant TSPO binding change at any time point. On day 7, there was a significant decrease in TSPO binding in the ION-CCI group relative to the naïve group within the midline cerebellar cortex. No change occurred in this region at any other timepoint in either the ION-CCI or sham-injury groups. On day 14, there were multiple regions where TSPO binding was significantly reduced in the ION-CCI compared with naïve groups. Binding decreases occurred in the region of the right septal nucleus, right hippocampus and in two clusters in the right caudate/putamen. These decreases did not occur at any other timepoint and in the sham-injury group only occurred at day 14 in the septal nucleus and one of the caudate/putamen clusters. Finally, on day 28, there were two regions that displayed increased binding in the ION-CCI compared with naïve groups. One cluster in the region of the right M1 that also increased at day 2, and another at day 28 only in the region of the right S1. The sham-injury group also displayed increased binding in these two regions only at day 28.

**Table 2:** Significant cluster properties including SUVr values in the whole brain analysis. * p < 0.05 voxel-by-voxel analysis. # p < 0.05, two sample *t*-test. Day 2: day 2; D7: day 7; D14: day 14; D28: day 28; ION-CCI: infra-orbital nerve chronic constriction injury; M1: primary motor cortex; S1: primary somatosensory cortex; SEM: standard error mean; SpV: spinal trigeminal nucleus; SUVr: standard uptake values ratio.

	**cluster size**	**t-value**	**day**		**SUVr** **mean ± SEM**	
				**naive**	**sham**	**ION-CCI**
*D2: ION-CCI > naive*						
right M1/SI	273	6.00	D2		1.30 ± 0.05	1.42 ± 0.03*
			D7	1.16 ± 0.03	1.33 ± 0.06	1.23 ± 0.03
			D14		1.14 ± 0.04	1.16 ± 0.04
			D28		1.37 ± 0.09	1.36 ± 0.05^#^
*D7: ION-CCI < naive*						
Right SpV	50	4.04	D2		1.63 ± 0.02^#^	1.64 ± 0.06^#^
			D7	1.49 ± 0.03	1.53 ± 0.05	1.68 ± 0.05*
			D14		1.42 ± 0.08	1.55 ± 0.04
			D28		1.40 ± 0.08	1.42 ± 0.02
cerebellar cortex	51	3.85	D2		1.60 ± 0.03	1.71 ± 0.09
			D7	1.63 ± 0.04	1.50 ± 0.04	1.41 ± 0.04*
			D14		1.56 ± 0.06	1.66 ± 0.07
			D28		1.75 ± 0.10	1.76 ± 0.05
*D14: ION-CCI < naive*						
right septal nucleus	38	3.59	D2		0.79 ± 0.01	0.78 ± 0.01
			D7	0.83 ± 0.02	0.77 ± 0.03	0.82 ± 0.02
			D14		0.75 ± 0.03^#^	0.73 ± 0.02*
			D28		0.93 ± 0.08	0.86 ± 0.04
right hippocampus	27	3.69	D2		1.07 ± 0.02	0.97 ± 0.02
			D7	1.03 ± 0.01	0.96 ± 0.02^#^	0.99 ± 0.02
			D14		0.99 ± 0.03	0.94 ± 0.01*
			D28		0.97 ± 0.03	1.00 ± 0.02
right caudate/putamen 1	26	3.71	D2		0.90 ± 0.02	0.85 ± 0.02
			D7	0.88 ± 0.01	0.85 ± 0.02	0.86 ± 0.02
			D14		0.79 ± 0.02^#^	0.78 ± 0.01*
			D28		0.93 ± 0.05	0.90 ± 0.02
right caudate/putamen 2	22	3.50	D2		0.75 ± 0.03	0.74 ± 0.02
			D7	0.77 ± 0.02	0.73 ± 0.02	0.75 ± 0.02
			D14		0.69 ± 0.03	0.66 ± 0.02*
			D28		0.84 ± 0.05	0.80 ± 0.02
*D28: ION-CCI > naive*						
right M1	55	4.12	D2		1.11 ± 0.06	1.24 ± 0.03^#^
			D7	1.02 ± 0.03	1.09 ± 0.03	1.06 ± 0.03
			D14		0.97 ± 0.03	0.99 ± 0.03
			D28		1.25 ± 0.11^#^	1.23 ± 0.04*
right S1	373	4.12	D2		1.16 ± 0.02	1.18 ± 0.04
			D7	1.07 ± 0.03	1.15 ± 0.05	1.09 ± 0.03
			D14		1.06 ± 0.02	1.03 ± 0.03
			D28		1.21 ± 0.04^#^	1.27 ± 0.03*

## Discussion

In this study our specific aim was to use PET imaging to visualise in-vivo, the activity of macrophages and microglia during the development of trigeminal neuropathic pain in male rats. Using chronic constriction injury of the infraorbital nerve (ION-CCI) (Vos and Strassman, 1995) we aimed to describe temporal changes in TSPO binding 2, 7, 14, and 28 days after ION-CCI, and to compared this to the binding in both sham-injured and naïve control rats. Unexpectedly, we identified almost identical changes in TSPO binding in rats that had undergone the *sham-injury* procedures, ie., the surgical isolation of the ION without ligation, to those seen in nerve injured rats. It is clear from these data that within this 28 day window, the surgical approach taken for nerve isolation, which involves skin incision and retraction of orbital contents is sufficient to evoke TSPO binding changes akin to those that are suggested to underly the development of the neuropathic pain state. The sham-injury effect that we have detected may share similarities to the model of persistent post-surgical pain triggered by skin-muscle incision and retraction (SMIR model) described in the hindlimb (Flatters, 2008). This procedure tiggers microglial activity in lumbar spinal segments attributed to the duration of exposure of the surgical site (Obata et al., 2006). We note however, that similar levels of microglial activity are not reported for sham-injury conditions in hindlimb neuropathic models, suggesting that sham-injury procedures in these instances are brief and perhaps more discrete, and likely that in preclinical trigeminal neuropathy models, the surgery for sham-injury is potentially more disruptive. We reflect that the *sham* condition is often considered a condition closer to the uninjured state, which is not always the case as surgical procedures still trigger significant tissue disruption with associated sensory hypersensitivity, as capitalised on in models of post-surgical pain. Further, we have recently described significant neuroimmune changes following identical sham-injury procedures on the infraorbital nerve (Kang et al., 2024) and so the fact that we detected TSPO binding changes in the sham-injured rats is perhaps not quite so surprising. We evaluated the main PET findings with immunohistochemistry using a combination of antibodies to identify TSPO binding sites (anti-PBR), combined with markers for glial cells and macrophages. Our immunofluorescence results indicated that in both infra-orbital nerve injured, and sham-injured rats, TSPO was likely binding to microglia, macrophages, astrocytes, vascular endothelial cells, as well as other non-identified classes of cell. Therefore, changes in TSPO signal are unlikely to reflect only macrophage accumulation or changes in glial cells. Secondly, we found that TSPO binds to specific sites along the trigeminal pain pathway at distinct timepoints. In addition, we found changes in TSPO binding in additional central sites including the cerebellum, the motor and somatosensory cortices, septum, hippocampus, and the striatum. To reduce the testing burden for both ethical and practical reasons, in this study we did not evaluate the rat’s behavioural responses to facial stimulation. We are however confident of the overall trajectories of both nerve injury-evoked, and sham-injury evoked changes in behaviour, based on data from a large-scale study that was conducted concurrently, and recently published (Kang et al., 2024). We consider the timing and location of the TSPO binding changes with regards to the changes described in that study and current literature. There is broad agreement that there are three distinct phases in the response to injury of the infraorbital nerve, these are characterized by both behavioural and neuroimmune markers. The first of the phases, described as the “early” period, lasts for up to one week, it correlates with the period of Wallerian degeneration and is characterized by hypo-responsiveness (Vos et al., 1994, Kang et al., 2024, Korczeniewska et al., 2017, Deseure and Adriaensen, 2004, Deseure and Hans, 2018). The second phase appears to be an “intermediate”, or transition period (12–21 days post-injury) where behavioural reactivity increases and animals show aversive or defensive responses to tactile stimulation (Kang et al., 2024, Korczeniewska et al., 2018). The final or “late” phase appears after 28 days and was described by Vos and colleagues (Vos et al., 1994), to last at least 130 days post ION-CCI and is characterised by well-established hyper-responsiveness and clear reductions in facial grooming. We will consider our PET findings using this temporal framework of ION-CCI evoked changes, from the site of injury and then at each level of the trigeminal pain pathway. As highlighted above, TSPO was selected so that we could identify changes in microglia and macrophages, however it is important to note however that we need to broaden this consideration to include reactive astrocytes, vascular endothelial cells, and possibly smooth muscle cells and neurons (Emvalomenos et al., 2024, Betlazar et al., 2020, Gui et al., 2020, Lavisse et al., 2012, Wimberley et al., 2018).

### Trigeminal Pain Pathway: Trigeminal Ganglion:

At the site of nerve injury, our histological analysis showed a significant accumulation of macrophages with “frothy” and vacuolated appearances, some of these cells colocalized with PBR-IR, indicating a likely site for TSPO binding. Following sham-surgery, there were small numbers of macrophages at the site of nerve exposure on each of the days of testing. PBR-IR was also colocalized with the endoneurial vasculature, we expect that this would be largely attributed to staining of the vascular endothelial cells. PET scans did not detect significant TSPO binding at the nerve-injury site at any timepoint. TSPO binding in the trigeminal ganglion was increased at day 14 in both the ION-CCI and sham-injury groups when compared to naïve rats. We also noted an increase in PBR-IR at this time point, however the numbers of CD68-IR macrophages in the ganglion were quite low and few of these contained PBR-IR. This raises the likelihood that the TSPO binding is not an indicator of macrophage accumulation. We noted that PBR-IR was present in the lumen of TL + vessels raising the possibility that TSPO was binding to endothelial cells, as well as other non-identified classes of cells. Binding of TSPO to endothelial cells has previously been reported in a mouse model of CRPS triggered by tibial fractures (Cropper et al., 2019). It has been reported that nerve injury can lead to increased vascularization of ganglia associated with the damaged nerve (LaMotte and Ma, 2008). These vessels can play a critical role in the development of neuropathic pain, through local release of inflammatory mediators such as cytokines and chemokines that directly, and indirectly alter the function of the resident satellite glial cells and sensory neurons (Willis et al., 2008, Kutcher et al., 2004, Pannese and Procacci, 2002, Han and Suk, 2005). The day 14 timepoint at which these observations were made is during the *transitional* phase, during which we have previously observed decreased numbers of macrophages relative to the acute injury phase, i.e., 2–7 days post ION-CCI, and prior to an apparent second wave of macrophage accumulation seen during the late phase, which includes 28 days post-injury and beyond (Kang et al., 2024). This transitional phase may well reflect the change from a phase of clearance of injury related cell damage, to a later phase of nerve repair and attempted functional recovery. Our observations in sham-injured rats suggest that the surgical procedures for nerve injury might also contribute significantly to these changes.

### Trigeminal Pain Pathway: Brainstem Trigeminal Complex:

Similar to the trigeminal ganglia, in both nerve-injured and sham-injured rats, PBR-IR was co-localized with the neurovasculature along the rostro-caudal extent of the brainstem trigeminal complex (*pars interpolaris*, *pars caudalis*, and Pa5) and the NTS, as well as in astrocytes and microglia. As such, the changes in TSPO binding that we observed along the brainstem do not appear to correspond to a single cell type, rather it emphasizes the close relationship between glial activity and the neurovasculature. Astrocytes are well known to regulate neuronal function and directly interact with the vasculature as well as other glial cells (Haydon and Carmignoto, 2006, Goenaga et al., 2023).

Increased TSPO binding was observed in a discrete region of the brainstem trigeminal complex ipsilateral to the injury, and surgical site, this region included the Pa5, the dorsal portion of SpV (*pars interpolaris* and *pars caudalis*), and the trigeminal portion of the NTS. These areas have all been described to receive direct inputs from fibres of the infraorbital nerve specifically (Panneton and Burton, 1981, Jacquin and Rhoades, 1983) and more generally from the maxiliary division of the trigeminal nerve. The paratrigeminal nucleus is a small interstitial system of the spinal trigeminal tract, which consists of small diffuse nuclei located in the dorsal lateral medulla (Chan-Palay, 1978). It receives sensory inputs not only from the trigeminal nerve, but also from the glossopharyngeal and vagal nerves (Ciriello et al., 1981, Marfurt and Rajchert, 1991) and is involved in the integration of both cardiovascular, and respiratory responses, in addition to pain mechanisms (Yu et al., 2002, Yamazaki et al., 2008, Yu and Lindsey, 2003). Its cells send efferent projections to both the parabrachial nucleus, the nucleus of the solitary tract, lamina 1 of the *pars caudalis* of the SpV, and the ventroposterior medial nucleus of the thalamus (VPM) (Menétrey et al., 1987, Torvik, 1956, Saxon and Hopkins, 1998). Similarly, the NTS receives direct sensory input from the trigeminal nerve, the glossopharyngeal and vagal nerves as well as inputs from the superficial laminae of the SpV, *pars caudalis* (Marfurt and Rajchert, 1991, Menétrey and Basbaum, 1987). The *pars interpolaris* and *pars caudalis* of SpV also receive direct inputs from the infraorbital nerve, via both the maxillary and ophthalmic divisions of the trigeminal nerve (Jacquin and Rhoades, 1983). These anatomical distributions of sensory inputs correspond well with the effect size analysis that we report here, which revealed increased TSPO binding ipsilateral to the nerve-injury, and the associated surgical site in the dorsolateral aspect of the laminar part of the SpV, as well as the Pa5 region and the dorsal NTS (trigeminal portion). Our data suggest that the surgical procedures for ION-CCI contributes significantly to these changes based on our observations of significant binding in sham-injured rats.

The dynamic changes observed in TSPO binding in the brain over the experimental period were similar to those seen at the site of nerve injury, and the trigeminal ganglion and suggest a common phasic response profile to both nerve injury and sham surgery. The largest increases in TSPO binding were noted 2 days after nerve injury and sham surgery, within the *early response* phase. In rats, an initial and rapid microglial response in SpV *pars caudalis* has been observed after inferior alveolar nerve transection and peaks between 1–3 days (Okada-Ogawa et al., 2009) this response profile is also seen following ION-CCI (Shibuta et al., 2012, Latrémolière et al., 2008). We note, as discussed above, that such a microglial response is not reported in sham-injured counterparts in other studies of ION-CCI, although it is reported for the SMIR model of post-surgical pain, which our sham-injury procedure may more closely resemble. This microglial response is not related to an immediate (1–3 days) increase in neuronal activity, however 7 days after ION-CCI, phosphorylated ERK (pERK) expression, a marker of neuronal excitation, provides evidence of increased neuronal excitation in SpV *pars caudalis* (Suzuki et al., 2013, Okada-Ogawa et al., 2015). Similarly, cFos expression increases at 9–12 days after ION-CCI, again indicating an injury evoked increase in neuronal activity sometime after the microglial peak response (Vos and Strassman, 1995). Furthermore, at 7 days post injury the mean background discharge and after-discharge rates of wide dynamic neurons were significantly larger in ION-CCI rats in the superficial laminae of the *pars caudalis* (Suzuki et al., 2013). The delay between the peak microglial response and the increased neuronal activation may be a result of microglial modulation of the extracellular matrix, whereby the microglia degrade the perineuronal nets forming the matrix, which facilitates neuronal excitation in a similar fashion to that described following sciatic nerve injury and dorsal horn neurons (Tansley et al., 2022).

Glia-glial interactions can also result in the initiation and maintenance of chronic neuropathic pain. At 7 days post injury, although microglia are still present, their numbers are decreased, and the presence of GFAP-IR hypertrophic astrocytes are increased following trigeminal nerve transection (Piao et al., 2006, Kubíčková et al., 2020). Microglial derived factors such as C1q are known to increase astrocyte reactivity and subsequently nociceptive signaling 7 days after ION-CCI (Asano et al., 2020). TSPO binding at 14 days post injury appears to show a more elongated distribution along the rostro-caudal axis of the SpV, and extends into its deeper laminae, the binding in the dorsal NTS and Pa5 is maintained at this timepoint also. This timepoint corresponds to the *transitional* phase where it is suggested that the primary glial activity is that of astrocytes. GFAP-IR is sustained in astrocytes 14- and 21-days post trigeminal transection injury (Piao et al., 2006, Zhang et al., 2012) as well as ION-CCI (Dieb and Hafidi, 2013, Kubíčková et al., 2020). It is important to note that GFAP-IR astrocytes during this *transitional* phase are present across both superficial and deep laminae of *pars caudalis*. We note that in the literature, there are fewer studies of glial expression following trigeminal nerve injuries at these extended timepoints, with most studies focusing on 7–14 days post injury.

In our rats at 28 days post injury or sham-injury, the TSPO binding contracts along the rostrocaudal axis, and becomes most concentrated in the dorsolateral aspect of the SpV/Pa5 region and the dorsal NTS. At this *late* phase of injury, microglia are reported to be still present following trigeminal nerve transection, albeit in much smaller numbers, and GFAP-IR astrocytes are sustained but also in smaller numbers following trigeminal nerve transection and ION-CCI (Piao et al., 2006). The contraction of TSPO signal around the Pa5 region is intriguing as this subregion has been shown to be more sensitive to nociceptive-specific C-fibre inputs than the SpV, and is thought to drive the affective-emotional elements of pain via its outputs to midbrain structures including the parabrachial nucleus and the PAG (Okada et al., 2019).

Our analyses of TSPO using binding density versus effect size analysis revealed differences in peak signals within the trigeminal brainstem complex. While these signals all fall well within the boundaries of termination of the infraorbital nerve derived from anatomical tracing studies as illustrated in Fig. 4, the shift deserves comment. These differences may reflect limitations of the resolution of the PET technique, or the TSPO ligand in particular, or they may in fact reflect salient neurobiological features of ION-CCI and sham surgical procedures. Whether the locations with the greatest effect sizes contribute to the sensory and behavioural consequences of ION-CCI and sham-injury , when compared with the sites showing the statistically significant binding increases, warrants future investigation.

### Trigeminal Pain Pathway: Cortical Structures:

*Primary motor cortex (M1) and S1):* In the current study, we also observed increased TSPO binding in a variety of forebrain structures including S1 and M1, ipsilateral to the nerve injury and surgical site, in both ION-CCI and sham-injured rats. This was evident during both the *early* phase (**D2**) and *late* phase (**D28**) after both ION-CCI and sham-injury. While contrary to the classical view that ascending nociceptive pathways terminate exclusively contralaterally, there is substantial evidence for an ipsilateral nociceptive pathway transmitting trigeminal pain (Henssen et al., 2016, Nash et al., 2010). Our observations could suggest surgery and nerve injury-evoked cortical plasticity; this proposal is based on observations that sciatic nerve ligations trigger astrocyte activation in the S1 within the first week of injury, and that this correlates with changes in extracellular glutamate concentrations and dendritic spine turnover indicative of neuroplasticity (Kim et al., 2016). Of note are data from Loggia and colleagues, who have shown in humans with chronic back pain, that significant increases in TSPO binding are present in both the S1 and M1 which provides strong evidence for prolonged glial activity that persists long after the initial injury (Alshelh et al., 2022, Loggia et al., 2015).

TSPO binding was also noted in a region of the midline cerebellar cortex, at approximately lobules 4 and 5 of the anterior lobe. This region receives direct sensory projections from the trigeminal nerve via the superior cerebellar peduncles (Marfurt and Rajchert, 1991), providing a route for injury and surgery mediated, direct modulation of glial-neurovascular contributions to cerebellar function.

*Sub-cortical Forebrain:* During the *transitional* phase (D14) the septal nuclei, hippocampus, and dorsal striatum showed decreased TSPO binding on the side ipsilateral to the nerve injury and sham-injury , consistent with the cortical changes described above. TSPO binding decreases are difficult to interpret, they may indicate a transient decrease in glial-neurovascular interactions. Altered metabolism, measured by [^18^F]fluorodeoxyglucose in awake rats has been shown at this timepoint in a range of subcortical forebrain structures following the spinal nerve ligation model of neuropathic pain (Kim et al., 2014) and striatal monoamine levels are significantly disrupted 13 days after a peripheral sciatic nerve CCI (Gemikonakli et al., 2019). A second possibility is nerve injury and surgery triggered mitochondrial dysfunction at specific central sites. TSPO is mitochondrial protein, to which [^18^F]PBR06 binds, decreased mitochondrial function/expression may lead to decreased [^18^F]PBR06 binding identified by regional reductions of the PET signal. It is of note that mitochondrial dysfunction is often associated with neuropathic pain-like symptoms (van den Ameele et al., 2020). Both of these suggested changes likely correlate with disruptions to both cognitive and affective behaviours, resulting for example, in impaired decision making.

## Conclusion

We provide *in vivo* evidence of transient increases in TSPO binding in the trigeminal ganglion and trigeminal brainstem complex following both infra-orbital nerve constriction injury and sham-injury procedures that isolate the ION in identical fashion, but do not ligate it. The use of *in vivo* imaging of glial cells in combination with other imaging techniques such as structural and functional magnetic resonance imaging, can provide a novel view of the complex interactions between neural and non-neural cells in preclinical models of neuropathic pain, and as suggested by observations in sham-injured rats in this study, in models of persistent post-operative pain. Given that these *in vivo* techniques can be used to acquire similar information in humans, our results provide the platform to begin to translate findings from preclinical models into human patients with chronic pain or the risk of persistent post-surgical pain and offer an opportunity to begin to explore the effects of treatments on neural and non-neural cells in both preclinical models and humans.

## Declarations

### Ethics approval and consent to participate

All experimental procedures were carried out with the approval of Alfred Medical Research and Education Precinct Animal Ethics Committee at the Precinct Animal Centre (PAC; E/8283/2022/M) and in accordance with the guidelines of the Code for the Care and Use of Animals in Research Australia, and Ethical Guidelines for Investigation Association for the Study of Pain (Zimmermann, 1983).

### Consent for publication

this manuscript does not contain any individual person’s data in any form.

### CRediT authorship contribution statement

**Gaelle M. Emvalomenos:** Writing – review & editing, Visualization, Investigation, Formal analysis, Data curation. **James W.M. Kang:** Writing – review & editing, Writing – original draft, Visualization, Investigation, Formal analysis, Data curation. **Sabrina Salberg:** Writing – review & editing, Investigation, Data curation. **Crystal Li:** Writing – review & editing, Investigation, Data curation. **Bianca Jupp:** Writing – review & editing, Resources, Methodology, Conceptualization. **Matthew Long:** Resources, Methodology. **Mohammad B. Haskali:** Resources, Methodology. **Sunil Kellapatha:** Resources, Methodology. **OIivia I. Davanzo:** Visualization, Investigation. **Hyunsol Lim:** Visualization, Investigation. **Richelle Mychasiuk:** Writing – review & editing, Supervision, Resources, Conceptualization. **Kevin A. Keay:** . **Luke A. Henderson:** Writing – review & editing, Writing – original draft, Supervision, Resources, Project administration, Methodology, Funding acquisition, Formal analysis, Data curation, Conceptualization.

## Funding

This work was supported by funding from the Australian National Health and Medical Research Council (Grant ID 1130280).

## Declaration of competing interest

The authors declare that they have no known competing financial interests or personal relationships that could have appeared to influence the work reported in this paper.

## Data Availability

The datasets used and/or analysed during the current study are available from the corresponding author on reasonable request.
