# Supplementary material for: Evidence for glial reactivity using positron-emission tomography imaging of translocator Protein-18 kD [TSPO] in both sham and nerve-injured rats in a preclinical model of orofacial neuropathic pain
Source: Neurobiol Pain. 2024 Dec 12;17:100175. doi: 10.1016/j.ynpai.2024.100175 (PMC11699482; doi:10.1016/j.ynpai.2024.100175)
Supplement: Supplementary Data 1 [file mmc1.docx]

**Supplementary Figure 1:** Representative photomicrographs of PBR-IR co-localization. (A) stitched 40x magnification photomicrograph of a ligated infraorbital nerve and corresponding with PBR-IR (orange), CD68-IR (green) and TL (red) co-localization with immunofluorescence. The PBR-IR in the infraorbital nerve of (B) naïve, (C) sham and (D) ION-CCI rats are depicted at x600 magnification. Example of co-localization of (E) CD68-IR and PBR-IR, (F) TL+ and PBR-IR and (G) CD68-IR, PBR-IR and TL+. White arrows depict co-localized immunofluorescent signals. Scalebars represent 20µm or 500µm in panel A.
